# Proactive Risk Assessment of Blood Transfusion Process, in Pediatric Emergency, Using the Health Care Failure Mode and Effects Analysis (HFMEA)

**DOI:** 10.5539/gjhs.v7n1p322

**Published:** 2014-12-26

**Authors:** Reza Dehnavieh, Hossein Ebrahimipour, Yasamin Molavi-Taleghani, Ali Vafaee-Najar, Somayeh Noori Hekmat, Hamid Esmailzdeh

**Affiliations:** 1 Research Center for Health Services Management, Institute for Future Studies in Health, Kerman University of Medical Sciences, Kerman, Iran; 2 Health Sciences Research Center, Department of Health and Management, School of Health, Mashhad University of Medical Sciences, Mashhad, Iran; 3 Student Research Committee, School of Health, Mashhad University of Medical Sciences, Mashhad, Iran; 4 Research Center for Modeling in Health, Institute for Futures Studies in Health, Kerman University of Medical Sciences, Kerman, Iran

**Keywords:** risk assessment, blood transfusion, Healthcare Failure Mode and Effects Analysis (HFMEA), emergency, pediatric

## Abstract

**Introduction:**

Pediatric emergency has been considered as a high risk area, and blood transfusion is known as a unique clinical measure, therefore this study was conducted with the purpose of assessing the proactive risk assessment of blood transfusion process in Pediatric Emergency of Qaem education- treatment center in Mashhad, by the Healthcare Failure Mode and Effects Analysis (HFMEA) methodology.

**Methodology:**

This cross-sectional study analyzed the failure mode and effects of blood transfusion process by a mixture of quantitative-qualitative method. The proactive HFMEA was used to identify and analyze the potential failures of the process. The information of the items in HFMEA forms was collected after obtaining a consensus of experts’ panel views via the interview and focus group discussion sessions.

**Results:**

The Number of 77 failure modes were identified for 24 sub-processes enlisted in 8 processes of blood transfusion. Totally 13 failure modes were identified as non-acceptable risk (a hazard score above 8) in the blood transfusion process and were transferred to the decision tree. Root causes of high risk modes were discussed in cause-effect meetings and were classified based on the UK national health system (NHS) approved classifications model. Action types were classified in the form of acceptance (11.6%), control (74.2%) and elimination (14.2%). Recommendations were placed in 7 categories using TRIZ (“Theory of Inventive Problem Solving.”)

**Conclusion:**

The re-engineering process for the required changes, standardizing and updating the blood transfusion procedure, root cause analysis of blood transfusion catastrophic events, patient identification bracelet, training classes and educational pamphlets for raising awareness of personnel, and monthly gathering of transfusion medicine committee have all been considered as executive strategies in work agenda in pediatric emergency.

## 1. Introduction

Patients’ safety is one of the main issues in a health care system and of high importance in hospitals due to the economic, humanitarian and ethical aspects, which are the first and the most important issues in professional identity (Pourreza, Akbarihaghighi, & Khodabakhshnejad, 2006). Clinical failures are considered as a serious problem in a health system and a threat for patients’ safety (Dehnavieh et al., 2013). These failures may happen during all the stages of diagnosis and treatment, and mostly impose an extra cost on health system and reduce the quality of life of patients (Adachi & Lodolce, 2005; Nasiripour, Raeissi, Tabibi, & Keikavoosi, 2010). Although eliminating clinical failure is not entirely achievable, avoiding it is a key component of health care quality. And patients’ safety programs are aimed to just minimize failures and reduce harm to patients (Clark, Meyers, Frye, McManus, & Perlin, 2012).

A risk management program in one of the hospitals in Melbourne, reduced the undesired events to hospitalized patients from 1.35% to 0.74% (61% decreases) and to emergency patients from 3.26% to 0.48% (78.2% decreases) (Wolff, Bourke, Campbell, & Leembruggen, 2001) Based on the Joint Commission on Accreditation of Health Care Organizations statistics, blood transfusion failures (2.9%) have been enlisted as the priority adverse events in all the US hospital wards including emergency wards, since 2008. Linden study showed that the incorrect blood transfusion procedure resulted in 1600000 deaths in New York in 2000 (Adibi, Khalesi, Ravaghi, Jafari, & Jeddian, 2012). Also the report of US Agency for Healthcare Research and Quality in 2007 showed that the number of blood transfusions has increased from 1.1 million to 2.7 million cases from 1997 to 2007 (Advis, 2010). Assessment of the existing risk in a system is the first step in eliminating or reducing the medical failures. In the past, the risk management programs in health sector had a reactive nature with the goal of solving the existing problems (Card, Harrison, Ward, & Clarkson, 2012). Since 1990, different proactive risk management programs were introduced, following the infusion of “Systems Thinking” which considered optimizing the systems and work processes as the best way to reduce failures by humans that are fallible (Card, Ward & Clarkson, 2012) According to the VA National Center for Patients’ Safety and the American Joint Commission on Accreditation of Health Care Organizations, HFMEA is one of the most leading risk management programs (Derosier, Stalhandske, Bagian, & Nudell, 2002). HFMEA is a systematic and proactive approach to identify and prevent the medical failures before they occur, and is widely used in the health care system (Cheng, Chou, Wang, Lin, Kao, & Su, 2012). By HFMEA approach, we can use a mechanism for categorizing the failure modes, identifying the root causes of failures and detecting the susceptible area in blood transfusion procedure for the purpose of increasing awareness of the staff and patients’ safety in this procedure. (Sorra et al., 2008)

Emergency ward is one of the most important wards in each hospital, performance of which has a great impact on the performance of other wards, and patient’s satisfaction (Zohoor & Pilevar Zadeh, 2003).

Approximately one third of patients referring to hospitals are for the pediatric emergency wards, and this ward has been identified as a high risk area in health care system (Wente, 2013; Bleetman, Sanusi, Dale, & Brace, 2012). Blood transfusion, as a unique clinical measure, is an important part of medical care that if properly used, can be life-saving (Tabiei, Nakhei, Saadatjo, & Taghobi, 2002).

Despite quality control systems and electronic data processing devices, still, miss transfusion is considered as a significant problem in blood transfusion medicine and a threat to patients’ life; it also imposes a huge cost to the health care system (Lippi & Plebani, 2011).

Given that preventing errors and improving patients’ safety in blood transfusion is a serious concern of health care centers, and considering that emergency ward is one of the main challenging areas of the health care field, and as transfusion of incompatible blood can endanger patient’s safety and life, therefore this study was conducted with the purpose of Proactive risk assessment of blood transfusion process in Pediatric Emergency of Qaem education- treatment center in Mashhad, by the Healthcare Failure Mode and Effects Analysis (HFMEA) methodology.

## 2. Methods

The study was a cross-sectional study with a mixed qualitative-quantitative method and is placed in applied research category based on its results.

Qaem Hospital is a general and first class hospital; having 815 active beds, 18 wards, 7 emergency wards, Para-clinic services and many medical clinics, and is placed in the level of one of the main educational-medical hospitals in the region and the country. This center acts as a medical research and training center for residency and post-doctoral students, in addition to providing medical care for the patients.

This research was conducted based on the five stages, defined in the methodology of HFMEA by the VA national center for Patients’ Safety, as follows (Derosier, Stalhandske, Bagian, & Nudell, 2002). In some cases, the steps have some differences in execution from the proposed model, due to the existing situation.

### First Step: Define the HFMEA Topic

Based on the expert’s views and assessment of the adverse events reported to the clinical governance office of the Qaem Hospital during the first 9 months of the years 2012, the blood transfusion procedure was selected to be analyzed for the HFMEA, to be worthy of manpower and time that is spent.

### Second Step: Assembling the Team

In this process, 11 individuals participated as the HFMEA team members. The multidisciplinary team included the responsible person for risk management (team leader), one expert in health care management (consultant), one supervisor, one assistant professor of emergency medicine, one resident, two nurses, one secretary, one laboratory technician, one laboratory expert and one blood bank officer (specialist team members).

### Third Step: Graphically Describing the Process

At this stage the blood transfusion diagram and its sub-processes were designed, modified and approved in a two-hour focus group discussion and personal interview, and was drawn on a flow diagram using VISIO software.

### Forth Step: Conducting Hazard Analysis which was done in 4 phases:

#### First Phase: Determining the Potential Failure Modes

All the failure modes for each of the processes and sub-processes of blood transfusion were enlisted via many brain storming sessions among team members. The list was transferred to the HFMEA forms after achieving consensus among team members. At this stage, all the failure modes were categorized based on the two models of “Proposed model for reducing the patients’ hospitalization duration” (Attar, To fighi, Hafezimoghadam, Maleki, & Goharinezhad, 2010), and “Classifying nursing errors in clinical management (NECM)” model (Tran & Johnson, 2010).

#### Second Phase: Determining the Hazard Score

The Hazard score was determined based on hazard scoring matrix (multiplying severity by probability of failure occurrence), and was registered in the HFMEA form sheets. The failures were categorized to four interventional levels “emergency, urgent, programming and monitoring” based on the hazard score in the hazard score matrix. (Bonfant et al., 2010) (Table 1).

**Table 1:** Hazard Score Matrix and Intervention Level

Severity Probability	Minor Event (1)	Moderate Event (2)	Major Event (3)	Catastrophic Event (4)	Intervention level
Frequent (4)	4	8	12	16	Emergency
Occasional (3)	3	6	9	12	Urgent
Uncommon(2)	2	4	6	8	Programming
Remote(1)	1	2	3	4	Monitoring

#### Third Phase: Drawing the Decision Making Tree

Drawing the decision making tree and deciding about how to proceed or stop in each of the failure modes is based on the three items of (Weakness point, Existing control and Detestability).

Forth phase: In this phase failure mode causes for each failure mode, where the decision is to “Proceed”, were recognized and categorized into 9 root causes using the consultative cause – effect meetings and in the format of an approved model by the UK National Health System (Vincent, 2003)

### Fifth Step: Action and outcome measures, which was done in two phases

#### First Phase: Description of Action

In this phase the proposed coping strategies for root cause in each failure mode was offered in the form of accept, control or eliminate.

#### Second Phase: Redesigning the Process

In this phase the improvement strategy for each failure mode was offered using the TRIZ (Akaya, Demırayb & Kurt, 2008) during the team meetings. Then feasibility of each strategy was considered, based on resources of the organization.

All the information related to the items of HFMEA forms was collected after achieving the consensus of team members by interviews (11 hours interview) and focus group discussions (five two-hour sessions; one session at the end of each stage).

## 3. Results

**Figure 1:**
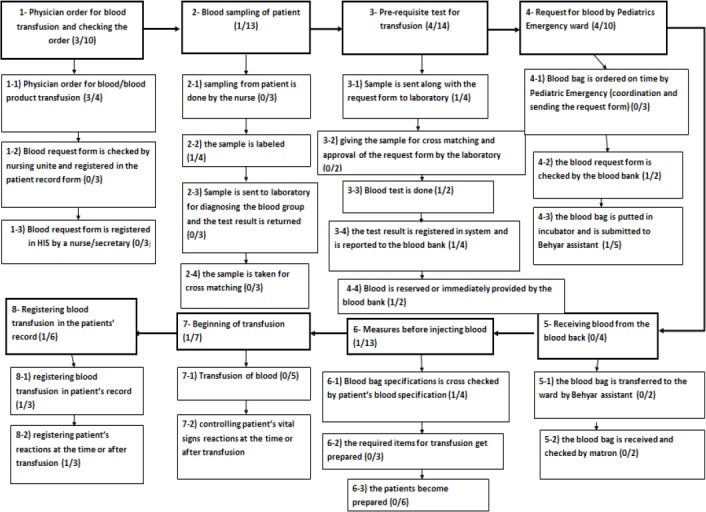
The graphical description of blood transfusion processes and the failure modes of each process step and process (number of high risk errors to the total number of errors)

Seventy seven failure modes were enlisted in the HFMEA forms, for the total 24 sub-processes of blood transfusion (figure 1). The percentage of each identified failure mode in each stage of the above mentioned processes to the total processes was: 12.9% was related to physician order for blood transfusion, 16.8% was related to blood sampling of patients, 18.1% was related to pre-requisite tests for transfusion, 12.9% was related to blood request by Pediatrics Emergency ward to the blood bank, 5.1% was related to receiving blood from the blood bank by the Pediatric Emergency ward, 16.8% was related to the required actions before injecting blood, 9.09% was related to the beginning of transfusion and 7.7% was related to registering blood transfusion in the patients’ record. In the other word, 49.3% of the failure modes were related to pre-analysis stage of blood transfusion, 14.28% to errors in the laboratory stage of blood transfusion and 36.3% to errors in the clinical stage of blood transfusion. 54.7% of failure modes were categorized in care process errors (17.8% clinical judgment errors, 21.9% clinical task execution errors, 2.7% technologies applied/ required errors and 12.3% continuity of care errors), 20.5% in communication errors (4.1% verbal communication errors and 16.4%written communication errors) 15.1% in administrative processes errors and 9.5% in knowledge and skill errors categories based on the classification of “Nursing Errors in Clinical Management”. Based on the “Proposed model for reducing the patients’ hospitalization duration” 27.2% of failure modes were categorized as mistakes, 29.8% as slip and laps, 19.4% as time errors and 23.3% were categorized as lack of the activity. Out of the 77 failure modes, 3 failure modes were placed in emergency level, 10 in urgent level, and 63 in programming level and one in monitoring level.

Totally, 13 failure modes were identified as high risk and unacceptable failures (risk score>8) in the blood transfusion process and were transferred to the decision tree. The root causes for unacceptable risk failure modes (discussed in cause-effect meetings) were organizational and strategic factors (12.7%), team factors (7.2%), patient factors(2%), individual factors (9.09%), task factors (18.1%), working condition factors (10.7%), equipment and resource factors (5.4%) communication factors (9.09%) and education and training factors (27.2%).

Recommendation actions for influencing factors of each error mode were presented in the form of accept (11.6%), control (74.2%), and elimination (14.2%). Finally, improvement strategies for each failure mode were offered using “TRIZ” and were classified in the form of training of guidelines and protocols (39.6%), developing scales for evaluation of performance and periodic evaluation and giving feedback to the personnel (22.4), reducing the staff work load and stress management (39.6%), regular meeting of blood transfusion committee for root cause analysis of catastrophic cases (8.6%), encouraging personnel to enquire about the ambiguities (12.06%), equipment management (5.1%) and reducing the inadequacy of manpower (3.4%) (Table 2).

**Table 2:**
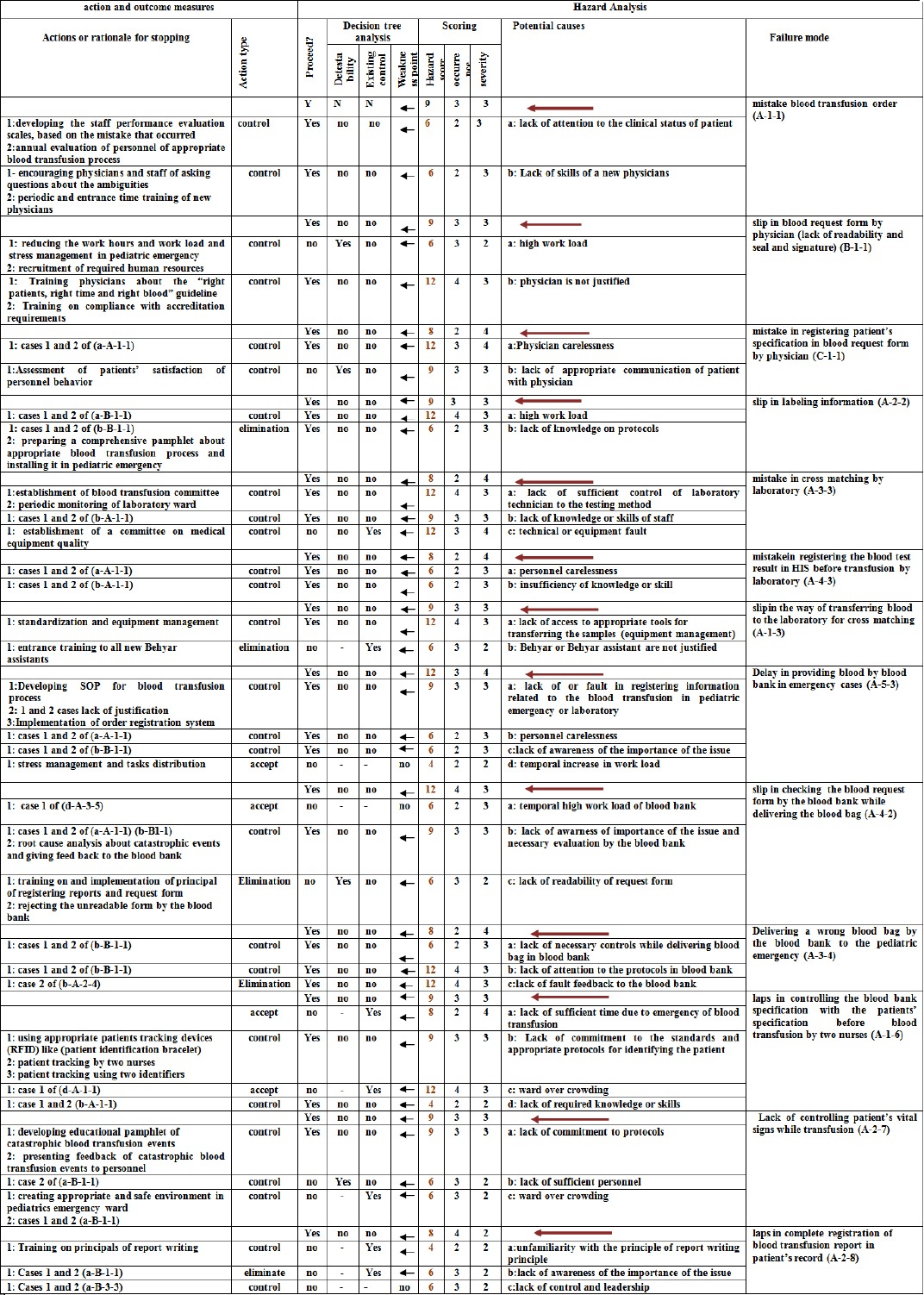
The HFMEA worksheet

## 4. Discussion

The present study was conducted with the aim of proactive risk assessment of blood transfusion process in Pediatric Emergency of Qaem education- treatment center in Mashhad, by the Healthcare Failure Mode and Effects Analysis (HFMEA) methodology.

Most of studies related to blood transfusion in health system have been done with a reactive approach, and have usually been done after occurrence of a catastrophic event. In this study, the authors used a proactive approach, “HFMEA”, to assess the possible failures of transfusion process, the influencing cause of each failure mode and to propose improvement strategies for transfusion processes.

Based on the results of Sorra and et al study, the main adverse outcome of blood transfusion can be avoided using preventive approach systems and failure management (Sorra et al., 2008). HFMEA has been identified as a proactive risk assessment tool that allows identifying of failure modes before a catastrophic event happened (Cheng, Chou, Wang, Lin, Kao, & Su, 2012). The number of medical adverse events has reduced from 3643 to 2412, after implementation of risk management programs including HFMEA by NCPS (Eadie, 2012)

In the present study, 54.7% of failure modes were categorized in care process errors, 20.5% in communication errors, 15.1% in administrative processes errors and 9.5% in knowledge and skill errors. In a study conducted by “Association of Management of Nursing Error” maximum failures were in the care process errors (66.6%), communication process errors (22.0%), administrative process errors (6%) and knowledge and skill errors (5%), which are not consistent with the present study (Tran & Johnson, 2010)

In the present study, 12.28% of failures were related to errors in laboratory and blood bank and 85.7% of transfusion errors were related to pre &post Analysis Stage of Blood Transfusion, which is consistent with the results of a study conducted in New York City, and with the results of Joint Commission on Accreditation of National Center for Patients’ Safety (Linden, Wagner, Voytovich, & Sheehan, 2000). In this study, the major transfusion failures were related to pre & post Analysis Stage of Blood Transfusion, which can be prevented with proactive measures.

Also according to the study of Pagliaro and Rebulla, most catastrophic events in blood transfusion happens in pre-analysis stage of blood transfusion (sample collection), among which, correct patients’ tracking consisted the highest percentage (Pagliaro & Rebulla, 2006)

In this study, the failure modes with the risk score equal or above eight were selected as unacceptable risks for the root cause analysis, which is consistent with most studies that used HFMEA technique (Cronrath et al., 2011)

One of the advantages of using HFMEA is prioritizing the influencing cause of each failure mode (Collins & Elsaid, 2010). In this study, using cause-effect analysis, the root causes of failures were classified, based on the UK NHS approved classifications model, into 9 categories. The maximum root cause of failures was related to the training factors (27.2%) and task factors (18.1%). In another study, the maximum root causes of failures were related to delay in service (51%) and inappropriateness of guidelines and protocols delivery (49%). As in the UK NHS approved classifications model, the inappropriateness of guidelines and protocols delivery is placed as sub-process of education/training items; therefore their results are consistent with the present study (Sorra et al., 2008).

The present study showed that 16.8% of identified failures required an improvement measure, and many of them were major events and without any appropriate controlling system. Dealing with such highly clear failures is very important. Therefore 39.6% of actions have been proposed in the field of developing guideline for correct conduction of blood transfusion procedure and periodic trainings of staff. Based on some reports, knowledge about the prevalence of adverse events in blood transfusion is available in most care centers, and only training is required to promote this knowledge among staff (Linden, Wagner, Voytovich, & Sheehan, 2000). Also, the Commission on Accreditation’s focus is on patient and blood sample identification in blood transfusion process, as its main objective in patients’ safety programs.

Considering the limited resources in each health care organization, the most cost-effective improvement strategies and approaches for eliminating the causes of failures and administering improvements should be selected. In this study, the proposed improvement strategies feasibility were discussed in team meeting sessions, based on the TRIZ approach, and, applying the re-engineering process for the required changes, standardization and updating the blood transfusion procedure, root cause analysis of blood transfusion catastrophic events, patient identification bracelet, training classes and educational pamphlets for raising awareness of personnel, and monthly gathering of transfusion medicine committee have been considered as executive among the proposed strategies in work agenda of Qaem Hospital. Aslani et al. also, in their study has considered establishment of blood transfusion committee as an effective measure for improving quality and training of staff (Aslani, Etemadyfar, & Noryan, 2004). In another study, focusing on simplifying the procedures and defining the key stages have been considered effective in appropriate conduction of blood transfusion procedures (Gray & Illingworth, 2013).

## 5. Conclusion

Using the proactive method of HFMEA for identifying the possible failure of treatment procedures, determining the effective cause on each failure mode and proposing the improvement strategies, has a high efficiency and effectiveness. Training and auditing should be considered as two main tools for improving the blood transfusion processes. Their application can reduce occurrences of failures and their outcomes to minimum possible level and provide a basis for quality improvement and risk reduction. Also, application of systematic and regular proactive risk management techniques, along with commitment of managers, and the organization policies renewal can ensure the effectiveness of these activities.

## Limitations

One of the limitations of this study is that the real failure level in studies based on HEMEA is not measurable and the scores given by team members are subjective; and based on the members’ position in hospital, the score given to a failure mode would differ. Therefore, individual interviews and surveys were done before focus group discussion sessions to prevent the group dominant effects in the team meeting.

The HFMEA lead to resource allocation to the problematic parts of the procedure (Van Tilburg, Leistikow, Rademaker, Bierings, & Van Dijk, 2006). On the other hand, determining the high risk failures in each institute is based on the organizational environment and cannot be compared to other organizations, because the frequency and severity of failures is not similar, even in similar wards in different hospitals.

In HFMEA studies, showing post-intervention reduction in adverse events is difficult like other qualitative strategies and one cannot prove the improvement of patients’ safety or do the cost-benefit analysis (Linkin et al., 2005).
